# Planning, preparing and structuring a small group teaching session

**DOI:** 10.1186/s12909-020-02281-4

**Published:** 2020-12-03

**Authors:** Christie van Diggele, Annette Burgess, Craig Mellis

**Affiliations:** 1 The University of Sydney, Faculty of Medicine and Health, The University of Sydney, Sydney, NSW 2006 Australia; 2 The University of Sydney, Faculty of Medicine and Health, Sydney Medical School, Education Office, The University of Sydney, Sydney, NSW Australia; 3 The University of Sydney, Faculty of Medicine and Health, Sydney Health Professional Education Research Network, The University of Sydney, Sydney, NSW Australia; 4 The University of Sydney, Sydney Medical School - Central, The University of Sydney, Sydney, NSW Australia

## Abstract

A structured approach is critical to the success of any small group teaching session; preparation and planning are key elements in ensuring the session is systematic and effective. Learning activities guide and engage students towards the achievement of agreed learning outcomes. This paper introduces the central concepts of planning and preparing a small group teaching session. It provides an overview of key theoretical principles in lesson planning, delivery, and how to provide effective feedback in this setting.

## Background

A small group teaching session that is well planned provides a systematic approach for both teachers and learners, whether it occurs in the university ‘classroom’, hospital or community ‘clinical setting’. Compared to didactic lectures, effective small group teaching and learning strategies increase student engagement, retention of knowledge, self-directed learning, communication skills, teamwork ability, and peer discussion [1–5]. Consequently, small group teaching has become increasingly popular within medical and health professions education. This paper introduces the central concepts of *planning and preparing* a small group teaching session. It provides an overview of the key theoretical principles in structure, lesson planning, different formats of small group teaching, delivery and provision of effective feedback to learners.

### Planning small group teaching

**Fig. 1:**
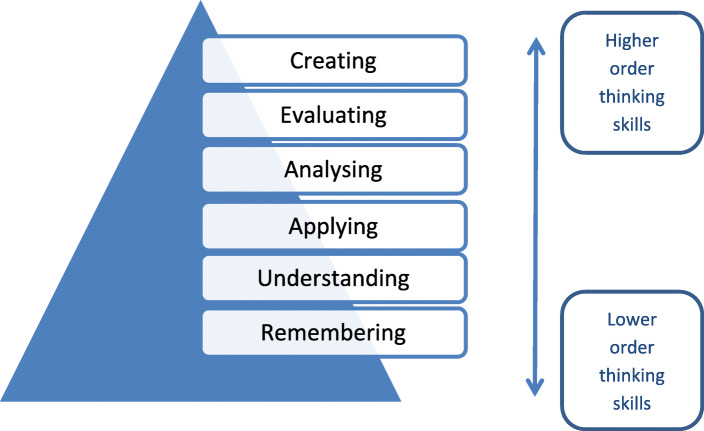
Bloom’s Taxonomy (adapted from Anderson et al., 2001 [6])

The planning of learning activities is an important part of course design and everyday teaching; curriculum and lesson design must be aligned in order to achieve the intended learning outcomes [6, 7]. Specifically, there should be alignment of the curriculum, the subject, learning outcomes, learning activities, and assessment tasks [6, 7]. Learning activities should encourage student participation and guide and engage students towards the achievement of set, agreed learning outcomes. They should also provide opportunities to: model thinking and learning strategies, practice skills, build on existing knowledge, learn from a range of sources (including peers) and gain feedback [7, 8]. *Bloom’s taxonomy* (Fig. 1) is a useful structure for lesson design. It is used as a tool for classifying lesson objectives and contains six categories that are structured in hierarchical order progressing in complexity as it reaches the highest point [6].

#### The learning cycle

**Fig. 2:**
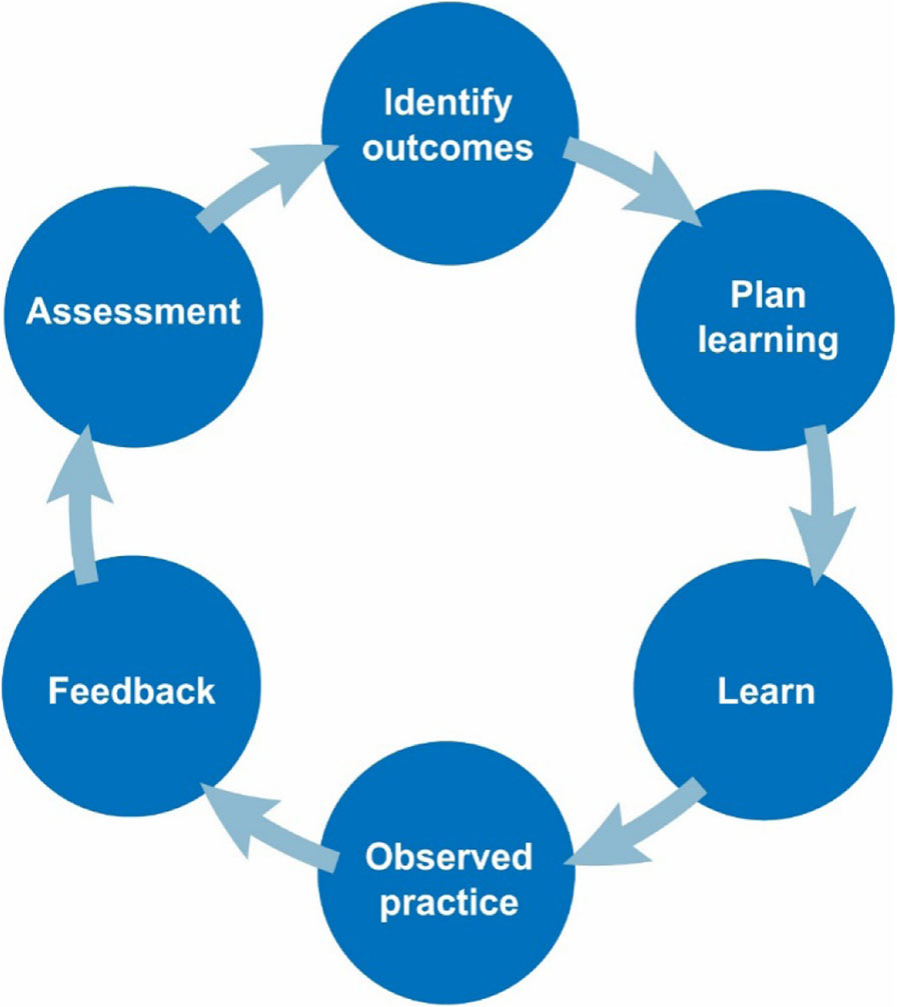
The learning cycle

The key characteristics of small group teaching are the active involvement of students in the entire learning cycle, and the interactive and social process. Within each pedagogy of small group teaching, students are encouraged to apply and transfer new knowledge through in-depth discussion, collaboration and reflection. This is referred to as “collaborative learning”, since it is centred around interactions between students, their peers and facilitators, rather than a one-way interaction, where knowledge is imparted from the teacher to the student. It is this social, interactive approach that lies at the centre of small group teaching. Planning forms a vital component of the learning cycle (Fig. 2) [8].

### Structuring a teaching session

Even though the clinical setting may be busy, it is possible to plan to teach common, recurrent topics, and follow a set structure. We propose the ‘Outcomes-Activity-Summary’ (OAS) method (Table 1) as a structure that can be applied for initial planning, whether in the classroom or clinical setting, when unexpected teaching and learning opportunities are more likely to be encountered. Two worked examples, where the OAS method has been applied to planning teaching sessions on ‘childhood asthma’ in the classroom setting (case-based learning) and clinical setting (bedside teaching) are provided in Table 2.

**Table 1:** Our proposed OAS method for lesson planning and teaching

	‘Outcomes-Activity-Summary’ (OAS)
**O**	**OUTCOMES**
	• Consider the background knowledge of students • Consider what you want the students to learn, understand, and be able to do by the end of the session • Establish your lesson goals and outcomes prior to the session • Share the learning outcomes with students at the start of the session • Allow the students input towards the learning outcomes
**A**	**ACTIVITY**
	• Design appropriate learning activities aligned with the outcomes • Plan the activities and how you will engage the learners • Plan the classroom environment and seating arrangements • Ensure students are active participants in the lesson • Ensure your lesson delivery is stimulating and you have the students’ attention • Address students by their name, ask questions and get everyone involved to check their understanding • If clients/patients are involved, gain consent before participation
**S**	**SUMMARY**
	• Ask students to identify one new point/knowledge/skill learnt • Summarise the content or skills covered • Complete the lesson with a take-home message and a self-directed learning task • Ensure the lesson finishes on time • Evaluate your own teaching and take on board feedback from students

**Table 2:** The OAS method for lesson planning and teaching

Examples of planning teaching sessions on childhood asthma using the OAS method	
***Classroom tutorial***	***Clinical bedside teaching session***
**OUTCOMES**	
*The session is designed for Year 1 medical students*. By the end of the session, students will be able to: • Identify and list the mechanisms of wheezing • Demonstrate a basic understanding of the pathophysiology of asthma • Identify different wheezing phenotypes in early childhood • List risk factors for development of asthma • Identify the requirements for lung function testing in airway obstruction, and especially its limitations in young children • Describe the principles of asthma management.	*The session is designed for Year 1 medical students*. By the end of the session, students will be able to: • Recognise the difference between wheeze and stridor • Recognise increased work of breathing • Distinguish normal breath sounds from wheeze • Understand that young children may be difficult (or impossible) to examine clinically • Be aware that parents may be unhappy for students to examine their child.
**ACTIVITY**	
*Case-based learning* (CBL) format will be used. (group of 6–10 students) • Students will be provided with pre-reading (eg. a journal article) • A clinical case will be used, providing history, physical and investigations. • Students will be required to work in their group to: − make a diagnostic decision based on the history, physical and investigations. − create a mechanistic flow chart linking the presentation (signs and symptoms) to basic mechanisms in order to explain the diagnosis. − create a management plan (a table with goals, options and qualifying factors).	*Bedside clinical tutorial* format will be used. (group of 5–6 students) • If appropriate, students will be asked to contribute to taking a structured history from the parent/s, and examine the child for signs of increased work of breathing, and auscultation of the chest to detect any abnormal breath sounds. • The availability of appropriate patients (a co-operative wheezing child) may limit achieving some objectives. Role play and other teaching methods may be necessary. • Flexibility with bedside teaching activities is essential, as parents may not consent to being utilised in teaching. • Tutors must be very aware of any perceived discomfort by patient and/or parents, and discontinue the bedside teaching.
**SUMMARY**	
• Given the high frequency of wheeze in young children, it is essential students have a good understanding of this common, important condition. • Note how early childhood wheeze differs from previous teaching on adults with chronic obstructive pulmonary disease (COPD).	• Wheeze and shortness of breath is a very common clinical presentation in early childhood, and it is essential students can recognise this condition and initiate appropriate management. • Ensure you look for signs of chronic illness such as failure to thrive/ poor weight gain.

### Designing a formal lesson plan

**Fig. 3:**
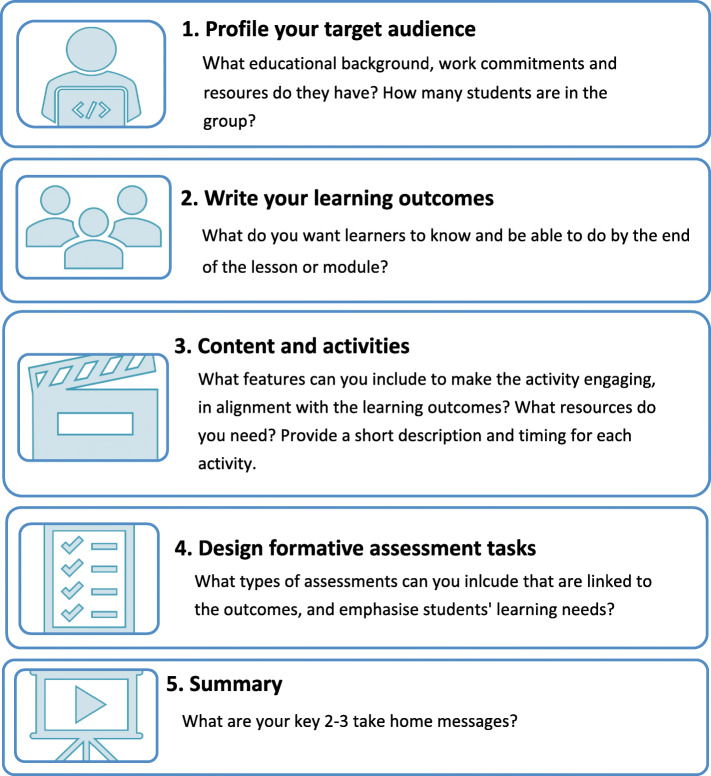
Five key steps to designing your lesson or module

A lesson plan acts as a map, assisting in guiding a series of activities to ensure students gain the knowledge, skills or attitudes set out within the learning objectives [9]. It also provides a record of what has been taught and assists in planning and alignment of assessment tasks. Although not all lessons can be planned, especially within the clinical setting, there are steps that can be taken to ensure a theoretical approach in lesson structure. An advantage of a lesson plan is that adjustments can be made to suit the needs of individual learners [9]. A lesson plan should identify the key aim and outcomes, content, structure and timing of activities and assessment tasks [7]. The five key steps to consider when writing a lesson plan are highlighted in Fig. 3 and described below [7, 8].

#### Profile of your target audience

Consider who will be participating in the lesson. Consider their background knowledge and learning needs. Also consider the resources that are available to you and to the learners, such as suitable, available patients.

#### Outcomes

Decide on focused and achievable learning outcomes for the teaching session [10]. Be clear (in preparation and in conveying to the learners) about learning outcomes, and what can be achieved in the limited time available. Make sure what you teach is relevant to the learners and pitched at the right level, considering the background knowledge of students. Consider environmental factors for the teaching session, such as seating arrangements and suitable lighting. If a patient is involved, make sure they are suitable, able and willing to participate. Although learning outcomes need to align with the curriculum, at the level of the individual teaching session, it is important to consider individual learning needs of participants. Ensure that agreement is reached on specific learning outcomes, which may require adjustment according to these needs and the teaching context [10]. Each lesson should have 3–6 learning outcomes.

##### Writing learning outcomes

Learning outcomes are the descriptors or goals students should know, or be able to do, at the completion of the session [7]. These may include knowledge, skills or attitudes. Whether devised by the teacher, or set by curriculum documents, outcomes should be provided to the students prior to, or at the beginning of the session, to ensure they know your expectations. Bloom’s taxonomy can be used to select appropriate verbs for the intended learning. In order to write learning outcomes there are three items to include [6]:

- a verb pitched at the appropriate stage of understanding or skill level,
- the content the verb is intended to address,
- the context in which the verb is to be deployed.

For example: A simple knowledge recall objective- *Explain* the importance of setting learning objectives. An objective that requires higher order thinking- *Evaluate* the importance of learning objectives.

#### Activity

This involves interaction between the teacher and learner/s. While the adult attention span is short (averaging at 10 to 20 min), active learning styles can significantly increase both attention span and knowledge recall in learners [6–8]. Ensure that you deliver the dialogue in a way that is brief, succinct, and relevant – that is, engaging. Address students by their names, ask questions to keep them actively involved, and to check their understanding. Consider including learning activities that vary and use students’ higher order thinking skills. With the current trend towards online learning, Learning Management Systems (LMS) can incorporate different types of content, including simulations, polls, quizzes, scenarios, animations, customised audio, interactive images, branching scenarios, videos, images, slides, and PDFs. Prepare the learning materials, organise the small groups, provide a clear explanation of the learning activities, and timing guidelines.

#### Assessment

Formative assessment provides a key driving force for learning [8]. It reinforces the information and skills learnt, and feedback should provide the learner with information on areas that may need improvement. In order for the assessment activity to be worthwhile learners need clear outcomes, an indication of their performance against these outcomes, and guidance on how to improve. Use effective questioning and assessment to keep learners actively involved throughout the lesson. Types of questions range from low level closed questions, to high order questions that go beyond simple recall, and engage the learner in problem-solving and critical thinking [11]. Through formative assessment tasks with feedback, learners can check their understanding, identify and address gaps in their knowledge. The learner’s interpretation of the feedback will direct and encourage self-regulated learning, where students monitor their own learning goals, and the strategies they use to achieve these goals [12].

#### Summary

Briefly summarise what has been covered in the session, and make links to previous learning. If you haven’t already, provide some feedback to students on their learning and any tasks that were done during the session. Ask students to identify the most important point/s, knowledge or skill/s that they have learnt during the session. Ensure you give two or three brief take-home messages, and advice on a self-directed learning task (i.e. an ‘educational prescription’). Make sure you finish on time.

### Types of small group teaching

The format of small group teaching activities required to develop the learners’ knowledge, skills and values needs to be considered in curriculum planning and teaching. A range of small group teaching methods have been developed, adopted and adapted within university medicine and health education curricula, according to available resources and student needs. Additionally, many online configurations of small group activities are emerging, particularly with the introduction of social distancing.

Popular methods for small group teaching in the university classroom setting are problem-based learning (PBL), case-based learning (CBL) and team-based learning (TBL), all providing learner-centred instructional approaches [2–4, 13]. Common to all three pedagogies, active learning with peer learning and discussion in small groups is based around a relevant, authentic clinical patient case; existing knowledge is activated; and new knowledge is applied to solve clinical problems [1–4, 13]. As outlined in Table 3, there are also some differences between these pedagogies.

**Table 3:** Overview of characteristics of PBL, TBL and CBL

Pedagogy	Brief overview of characteristics
**Problem-based learning** **(PBL)**	PBL is characterised by small group learning (6 to 10 students per group), using a guided learning format, with facilitation by one teacher. Learning takes place through problem-solving and self-study. Students initially meet to discuss the issues requiring further self-study, and then the group reconvenes to discuss and synthesise their learning. The facilitator is normally only present at the second meeting.
**Team-based learning** **(TBL)**	TBL is characterised by a format that permits one content expert to effectively facilitate a large number of small groups (for example, 12 groups of 6 students in one classroom), it uses a ‘flipped classroom’ technique, and a structured in-class learning format. TBL follows a sequence of steps, including pre-class preparation, in-class individual test and team-test, immediate feedback, and problem-solving activities.
**Case-based learning** **(CBL)**	CBL is characterised by small group learning (6–10 students per group), using an inquiry-based learning format, with facilitation by one teacher. Compared to PBL, CBL is less time consuming, and draws the focus of the students to key points of the clinical case. A structured and critical approach to clinical problem-solving is encouraged in CBL, where the facilitator is a content expert who directs and redirects the students.

While patient case discussions are common in healthcare education, other methods of engaging learners can be implemented within large class settings, to give students a small-group learning experience. Examples include [14]:

- *Paired discussion:* one-to-one discussion on an assigned topic for 3–5 min. The teacher is able to join in on the discussion
- *Break out groups:* the teacher poses a question and learners (in groups of 2–4) discuss responses before sharing with the whole group
- *Creation of posters/drawings:* for example, a mechanistic flow chart to describe the pathophysiology of the disease process
- *Group round:* generates interest in a topic with each learner having one minute to present their brief response. The order of participation can be selected at random and learners can pass their turn at least one time. For example asking for a ‘brief verbal synopsis’ of a clinical trial at a journal club, where each attempt will get progressively more succinct, clearer, and more accurate [14]
- *Brainstorming:* can produce a large number of creative solutions in a short period of time. This method encourages learner recall of knowledge and promotes interaction
- *Role play:* can be useful for developing communication skills e.g. interviewing. Sometimes actor patients/clients may be recruited for advanced role plays
- *Workshops:* a mixture of individual and group activities, with brief lectures
- *Seminars*: a report by students or a group of students, or discussion of a paper.

Small group teaching and learning formats in the hospital setting include bedside teaching, clinical tutorials, student-led tutorials [14] and SCORPIOs (Structured, Clinical, Objective References, Problem-based, Integrated and Organised) [15]. Within each of these contexts, there will clearly be differences in how small group learning is approached, even though the general principles are similar. Whether in the clinical or the university medical and health education setting, the goals of the small group teaching format are similar. Students want to be able to ask questions and ‘think things through’; check their understanding of material; work as a team and learn from each other; apply content to clinical or ‘real life’ situations; and learn to problem solve [16].

### Learning environment and seating arrangements

**Fig. 4:**
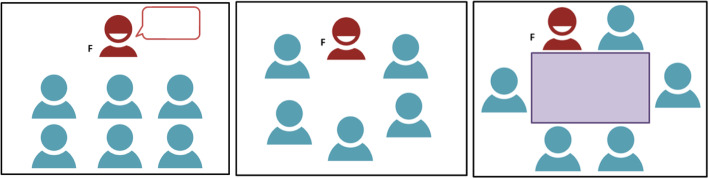
Lecture style seating, Group discussion seating, Discussion table seating (adapted from McKimm and Morris, 2009) [9]

Before the commencement of a small group teaching session, consider optimising the seating arrangement. Various seating arrangements have the potential to alter class discussion and interaction [9]. Each arrangement serves a different purpose and your selection should be based on the type of activity you are planning for your lesson. Below are a series of configurations (Fig. 4) depicting some common seating layouts for small group teaching sessions.

*Lecture style seating:* is a formal seating arrangement that is good for lecture style delivery, but does limit group interaction. It is clear that the facilitator leads the group with all of the chairs in rows facing the front of the room.

*Group discussion seating****:*** allows good group discussion, with the teacher forming part of the group. The teacher is seen as being equal rather than being in a leadership position. All group members have eye contact encouraging participation by all group members.

*Discussion table seating:* Although the table may act as a barrier for movement, this seating arrangement has the facilitator set within the group of learners. It provides space for working with papers/resources and encourages relaxed discussion between all members. However, some learners may feel less included because eye contact from the teacher may be limited with some seating arrangements.

### Delivery of the small group teaching session

Small group teaching design and delivery should be based on key principles that include the introduction to the topic; ground rules; group maintenance role and tasks role; activity; briefing, debriefing; and feedback [9, 13]. The flexible nature of small group learning means that the approaches can be tailored to meet the individual needs of students, and focus on the development of specific knowledge and skills. Effective facilitation allows students to develop not only content knowledge, but also critical thinking skills. Strategies to enhance the effectiveness of these teaching sessions include [16–18]:

1. *Coherence and flow:* the lesson should be linked through activities and content that relate and continue on from previously learnt content and skills
2. *Variety*: a certain degree of predictability should remain (e.g. teacher, environment), however, the lesson plan should range in activities and topics to ensure student engagement
3. *Flexibility:* an effective teacher needs to be able to think on their feet, and modify the lesson at any point to keep student interest, or follow unexpected questions.

There will always be diversity in learning preferences among students in any one group, and it is the facilitator’s role to assist all students to learn [7]. Consequently, teaching methods should be varied to cater for the different learners. Some learners will engage readily in learning activities, while others may be less motivated, and require greater guidance to form a deeper level of understanding, particularly where activities are specifically designed to use higher order thinking skills.

#### Feedback in the small group teaching setting

**Fig. 5:**
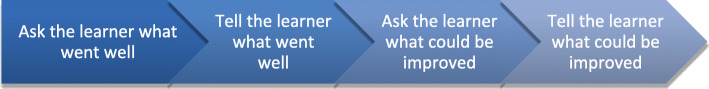
Feedback model (data from Pendleton et al., 1984) [18]

Provision of feedback helps close the gap between current and desired performance, and has the greatest impact on learning when it is immediate. Ensure that your teaching plan includes time for individual feedback to learners. Feedback can be provided by both peer learners and the facilitator. Feedback promotes learning by informing the student of their progress and the specific areas needing improvement; motivating the student to engage in relevant activities to further their learning; reinforcing good practice; and promoting self-reflection. Use of a structured method for feedback, such as Pendleton’s model [18], illustrated in Fig. 5, offers the learner the opportunity to evaluate their own performance, and prompts immediate feedback from the observer.

## Conclusion

In order to optimise learning and maximise engagement, teaching activities should follow a recognisable structure and ideally, be planned. Key issues to address when preparing for a small group teaching session include: determining the learning outcomes, designing the learning activities; aligning the learning outcomes with learning activities, the curriculum, and assessment; and ensuring seating arrangements optimise engagement. Students’ learning experiences should encourage active participation, opportunities for practice, and the provision of feedback.

### Take-home message

**Table:** 

• Successful teaching activities are well structured. • Use a structured format (such as the OAS method: ‘Outcome-Activity-Summary’) to plan and structure a small group learning session. • Clear learning outcomes, and alignment of activities and assessment are essential. • Provision of feedback is critical in ensuring learning is effective; formative assessment provides a structure for learner-centred feedback.	

## Data Availability

Not applicable.

## Abbreviations

PAL: Peer Assisted Learning
OSCE: Objective Structured Clinical Examination
OAS: Outcomes, Activities, Summary
CBL: Case-based learning
PBL: Problem-based learning
TBL: Team-based learning
SCORPIO: Structured, Clinical, Objective References, Problem-based, Integrated and Organised
COPD: Chronic obstructive pulmonary disease
